# Neural glycoprotein M6a is released in extracellular vesicles and modulated by chronic stressors in blood

**DOI:** 10.1038/s41598-017-09713-0

**Published:** 2017-08-29

**Authors:** Melisa C. Monteleone, Silvia C. Billi, Marcela A. Brocco, Alberto C. Frasch

**Affiliations:** Instituto de Investigaciones Biotecnológicas - Instituto Tecnológico de Chascomús (IIB-INTECH), Universidad Nacional de San Martín - Consejo Nacional de Investigaciones Científicas y Técnicas (UNSAM-CONICET), Av. 25 de Mayo y Francia, CP: 1650, San Martín, Buenos Aires, Argentina

## Abstract

Membrane neuronal glycoprotein M6a is highly expressed in the brain and contributes to neural plasticity promoting neurite growth and spine and synapse formation. We have previously showed that chronic stressors alter hippocampal M6a mRNA levels in rodents and tree shrews. We now show that M6a glycoprotein can be detected in mouse blood. M6a is a transmembrane glycoprotein and, as such, unlikely to be free in blood. Here we demonstrate that, in blood, M6a is transported in extracellular vesicles (EVs). It is also shown that M6a-containing EVs are delivered from cultured primary neurons as well as from M6a-transfected COS-7 cells. Released EVs containing M6a can be incorporated into COS-7 cells changing its phenotype through formation of membrane protrusions. Thus, M6a-containing EVs might contribute to maintain cellular plasticity. M6a presence in blood was used to monitor stress effects. Chronic restraint stress modulated M6a protein level in a sex dependent manner. Analysis of individual animals indicated that M6a level variations depend on the stressor applied. The response to stressors in blood makes M6a amenable to further studies in the stress disorder field.

## Introduction

M6a is a glycoprotein expressed in the neuronal membrane with a prominent expression in the brain. It participates in neurite outgrowth, filopodium formation and synaptogenesis^1–4^. Large-scale analysis on protein composition of peripheral fluids showed the presence of M6a glycoprotein in urine and in cerebrospinal fluid (CSF) of healthy human adults^5–7^. However, this protein has not been detected in blood so far. Since M6a is an integral membrane protein similar to the members of the tetraspanin family, in peripheral fluids, M6a might be transported associated to extracellular vesicles (EVs). These nano-sized vesicles are generated within the endosomal system or at the plasma membrane. EVs are shed from almost all cell types in both physiological or pathological conditions^8^. They are found in the extracellular space as well as in biological fluids. These vesicles carry specific RNAs, microRNAs, lipids and proteins from the cell they were released^8, 9^. EVs constitute another way of intercellular communication since they may function as vehicles for the transport of diverse cargo among different cell types^9, 10^. It has been demonstrated that primary neurons in culture release vesicles which can be isolated from conditioned medium *in vitro* ^11^ and also from CSF, confirming the *in vivo* production of neuronal-derived EVs^12^. Interestingly, the EV content may reflect the current cellular state^9, 13^, even during pathological processes, such as cancer^14^ or neurodegenerative diseases^15^. EVs have been isolated from post-mortem brain samples of patients diagnosed with schizophrenia and bipolar disorder. Compared to control samples, these EVs showed a different microRNA composition^16^. Differences in EV content between samples from individuals with or without mental illness suggests that the extracellular vesicles may contain markers for neuropsychiatric diseases^13, 15^.

Studies in animal models of psychosocial^17^, physical^18^ or prenatal stress^19^ showed that stress altered the M6a mRNA levels in the hippocampus. Antidepressant treatment restored M6a mRNA levels to that in the control animals^17, 18^. In humans, altered hippocampal M6a mRNA levels have been reported in post-mortem brain of depressed suicides^20^. Polymorphisms in the *GPM6A* gene sequence have been associated with pathological conditions such as schizophrenia^21^, bipolar disorders^22^ and claustrophobia^23^.

In this work, we reveal that M6a is present in peripheral fluids and show that in serum, M6a is transported as a protein in extracellular vesicles. EVs containing M6a were able to alter recipient cell phenotype, possibly by protein transfer. Furthermore, as a projection of M6a in EVs, we tested the hypothesis that chronic restraint stress affects peripheral levels of M6a protein. We demonstrate that blood M6a levels are modulated by stress in a sex-specific manner. Altogether this work provides evidence that glycoprotein M6a is present in EVs and its levels are modulated in blood after stressful events.

## Results

### Glycoprotein M6a can be detected in peripheral fluids

**Figure 1:**
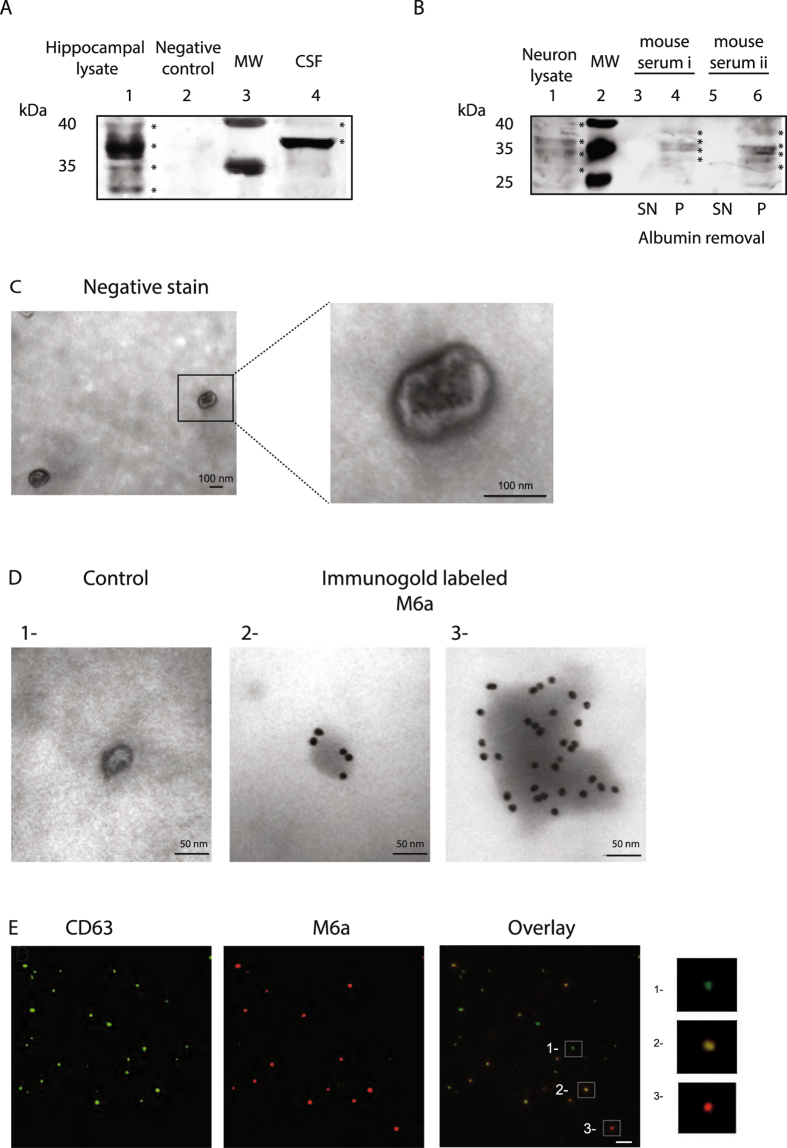
M6a is present in peripheral fluids. (**A**) M6a detection in rat cerebrospinal fluid (CSF). Lane 1: Hippocampal lysate, used as a positive control; Lane 2: COS-7 cells lysate (a cell line that do not express M6a endogenously), used as a negative control for M6a expression; Lane 3: Molecular Weight Marker (MW); Lane 4: rat cerebrospinal fluid. Bands representing M6a are indicated by stars. (**B)** M6a detection in mouse sera depleted from albumin protein. Two (i and ii) independent samples of two mice are shown. Lane 1: Neuron lysate, used as a positive control; Lane 2: Molecular Weight Marker (MW); Lanes 3 and 5: Supernatants (SN) obtained after albumin removal. These lanes show no M6a signal. Lanes 4 and 6: Corresponding pellets (P) obtained after albumin removal. Most minor proteins remain in pellets. Bands representing M6a are indicated by stars. As showed in **A** and **B**, M6a migrates as multiple bands due to post-translational modifications of the protein. M6a can be detected as four bands, as a doublet or as one intense single band depending on running conditions, protein load, membrane exposure and sample origin and processing. Full-length blots are shown in Figure S8. (**C**) EVs isolated from serum samples, observed by transmission electronic microscopy (TEM) negative staining. (**D**) Immunogold stain (18-nm particles) without (control, 1) or with monoclonal M6a antibody (2 and 3). Note the particles bound to the EV surface. A single EV (2) as well as a cumulus of EVs (3) are shown. (**E**) Serum-isolated EVs stained for the positive EV marker CD63 and revealed with a secondary antibody conjugated to Alexa-488 (green) or stained for M6a and revealed with a secondary antibody conjugated to rhodamine (red). Magnifications show a CD63-positive EV (green, 1- upper), a double labelled EV (orange, 2-﻿middle)﻿ and an M6a-positive EV (red, 3- lower).

Western blot assays were used to detect M6a in peripheral fluids. Rodent hippocampal tissue or neuron lysate, which contain high levels of M6a glycoprotein, were used as positive controls, not for quantification purposes (Fig. 1A and B). M6a appears as multiple bands that migrate between 35–40 kDa. These bands reflect M6a post-translational modifications such as phosphorylation and glycosylation (Fig. S1A, B)^24, 25^. Figure 1A,B show M6a detection in CSF and in serum samples, respectively. Due to the diverse sample complexity and to M6a concentration, bands detected in CSF and in serum samples may differ. Reactive bands of the expected size were clearly detectable in CSF (Fig. 1A). Albumin is such an abundant protein in serum that it does not allow the detection of minor proteins. Therefore, in the case of serum, samples had to be depleted of albumin prior to the assay in Western blots (Fig. 1B). Four bands were detectable in the pellet of albumin-depleted serum (Fig. 1B, lanes 4 and 6 show serum samples from two mice), bands which were hardly detectable in the supernatant (Fig. 1B lanes 3 and 5).

### M6a is released in extracellular vesicles

Since M6a is a membrane glycoprotein having four hydrophobic trans-membrane domains, we considered likely that this protein circulates in blood coupled to structures such as the extracellular vesicles (EVs). To test this hypothesis, we isolated by ultracentrifugation EVs from a pool of mouse sera. Figure 1C shows a transmission electron microscopy (TEM) image of EVs displaying the typical characteristics previously documented for EVs: size range between 30–100 nm and a round or a cup-shaped morphology^13, 26^. To provide evidence of the presence of M6a in these EVs, immunogold stain was carried out. When anti-M6a was incubated with the EVs, several 18 nm gold nanoparticles were bound to the EVs (Fig. 1D, panels 2 and 3). When the anti-M6a antibody was omitted, EVs were still observed, but devoid of gold nanoparticles (Fig. 1D, control, panel 1). To further characterize serum EVs, we analysed them by indirect immunofluorescence with antibodies against M6a and CD63. CD63 is a tetraspanin protein enriched in EVs and, therefore it is used as an EV marker^27^. We found that most EVs were positive for CD63 (Fig. 1E and magnification 1, upper). Some of these CD63 positive EVs contained also M6a, (Fig. 1E overlay and magnification 2, middle). Interestingly, we also found a population of EVs that carried M6a but not CD63 (Fig. 1E and magnification 3, lower). Our results show that different populations of EVs are present in serum and that M6a may be a marker of an EV subset.

**Figure 2:**
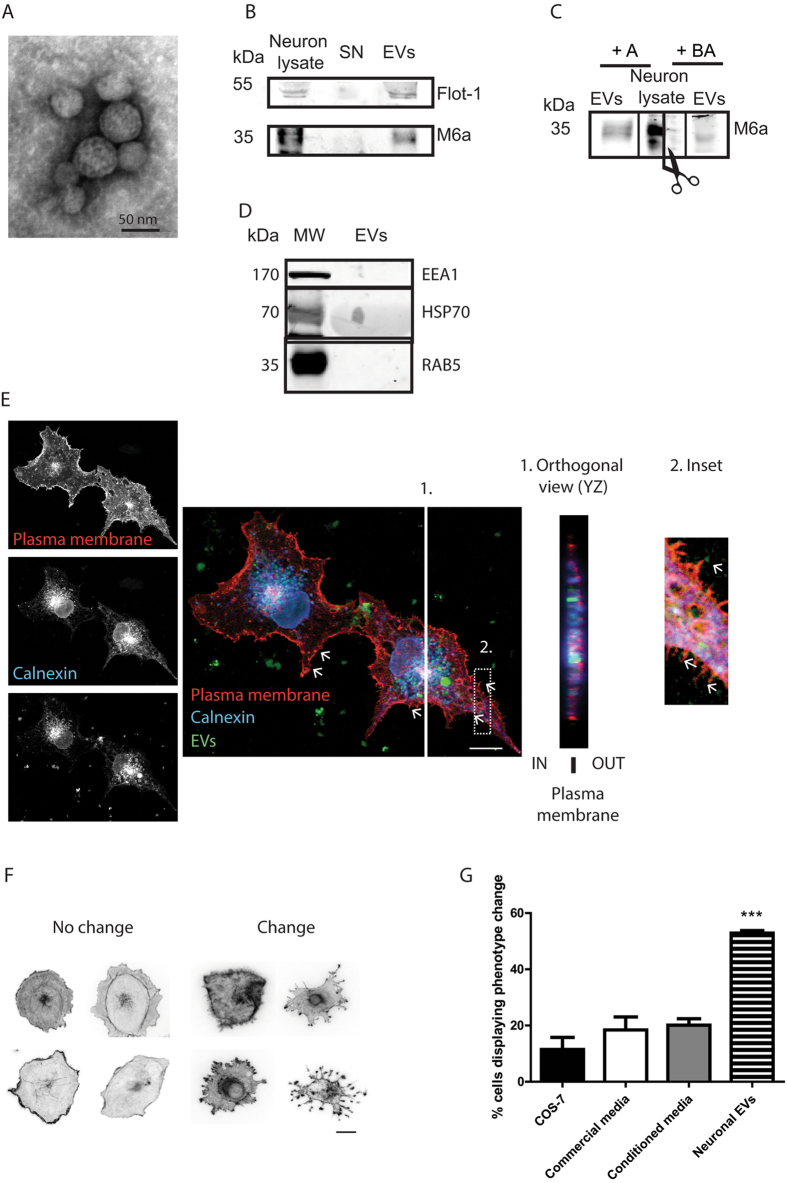
M6a is released into neuronal extracellular vesicles (EVs) that induce membrane protrusions in recipient cells. **(A**) Representative TEM images of EVs isolated by ultracentrifugation from culture medium of hippocampal neurons. During the sample processing for TEM analysis, some vesicles may collapse while others may not. This explains the perfectly round shape of the isolated EVs. (**B)** Western blot analysis of neuronal lysate (positive control), ultracentrifugation supernatant and pellet (containing the EV fraction) with the positive marker flotillin 1 (upper panel) and with M6a (lower panel). (**C)** Western blot reacted with anti-M6a antibody. Scissors represent a cut in the blotting membrane. The left side was incubated with anti-M6a alone; the right side of the membrane was incubated with anti-M6a in the presence of blocked antibody (BA). Note that the blocking peptide greatly reduces M6a signal. Full-length blots are shown in Figure S9. (**D**) Western blot analysis of neuronal. Negative EV markers used were EEA1 and Rab5. HSP70 was used as a positive EV marker. Similar amounts of protein (25 µg) were loaded in each lane. Western blot membrane was cut and incubated with the indicated antibodies. MW: Molecular Weight Marker. Full-length blots are shown in Figure S10. (**E**) Representative image of COS-7 cells transfected with the PH-delta-RFP construct whose protein product locates in the plasma membrane (left, upper panel); stained with calnexin (an ER marker; left, middle panel) and treated with DiO labelled EVs (left, lower panel). On the right panel, the overlay of the three channels is shown. Panel 1 shows a projected (YZ) orthogonal section along the white line in the image derived from 11 z-slices 0.4 µm apart. Arrows and inset (panel 2) show direct contact of EVs with membrane protrusions. Scale bar 20 µm. (**F)** COS-7 cell phenotype change after addition of neuronal EVs. Scale bar 10 µm. (**G)** Quantification of the percentage of cells displaying phenotype change. COS-7 cells were treated with: EVs isolated from non-transfected COS-7 cells (COS-7), defined medium from cultured neurons, medium from hippocampal neurons maintained *in vitro* for 14 days (conditioned media) or EVs isolated and concentrated from neuronal culture medium. n = 3 independent experiments, more than 100 cells counted per each condition. **P < 0.0001. ANOVA followed by Dunnet post-test.

Given that M6a is mainly expressed in neurons; these cells should be able to release EVs containing M6a. To evaluate this possibility, EVs were isolated from conditioned media from 14 days in vitro hippocampal neurons in culture. Different centrifugation steps were included to remove cells and cell debris (Fig. S2). Then, EVs were analysed by TEM and by Western blot. TEM negative staining revealed the presence of spherical, membrane encapsulated particles with diameters varying between 25–60 nm (Fig. 2A), consistent with previously described EV size^13, 26, 28, 29^. Western blot reacted with anti-M6a and anti-flotillin-1 (another EV marker). A neuron lysate was included in the blots as positive control. These antibodies showed that both proteins are present in the EV fraction and absent from the supernatant fraction (Fig. 2B) indicating an effective enrichment of them in the EV fraction. To evaluate anti-M6a antibody specificity, a blocking peptide was used. These assays showed a significant reduction on M6a stain in both neuron lysate and EV fraction (Fig. 2C, see also Fig. S1C). Altogether these results confirm that hippocampal neurons produce EVs that contain M6a and could, therefore, be one of the sources of peripheral M6a. EVs were also reacted with the negative markers for EVs such as the endosome marker early endosome antigen 1 (EEA1) and the early endosome marker Ras related protein 5 (RAB5). The chaperone heat shock protein 70 (HSP/HSC70) was used as a positive marker. Western blot analysis showed that both EV negative markers were present in cell lysates but none in the EV fractions (Fig. 2D). As expected, HSP70 was detected in the EV fraction.

### M6a coupled to EVs promotes membrane protrusion formation in recipient cells

Next, a possible role of M6a in the EVs was tested. It was previously shown that M6a has the ability of inducing membrane protrusions in primary culture of neurons as well as in non-neuronal cells such as COS-7 cells^1^. Therefore, we analysed if EV-coupled M6a retained this ability. EVs isolated from neuronal cultures were added (50 µg EV protein/8 × 103 cells/72 h, conditions standar﻿dised in previous experiments, Fig. S3) to sub confluent COS-7 cells that do not express M6a. The uptake of these EVs by COS-7 cells was evaluated by confocal microscopy (Fig. 2E). EVs were stained with the lipophilic dye DiO (green). As control, the supernatant from the DiO staining procedure as well as PEG precipitated DiO were incubated with cells to show that residual free DiO do not stain cells (Fig. S4). To visualise COS-7 cell membrane, cells were transfected with a plasmid coding a phospolipase C (PLC) domain fused to the red fluorescent protein (RFP, see Supplementary Information for further details). Calnexin, an endoplasmic reticulum (ER) marker, stain is seen in blue. Colocalisation of EVs with ER and the direct contact of EVs with cell membrane protrusions indicated vesicle internalisation (Fig. 2E, arrows and inset). The YZ orthogonal view confirmed that EVs localised within the cell (Fig. 2E). Neuronal EVs induced dramatic phenotypic changes in recipient COS-7 cells. Membrane protrusions of different length and morphology were observed on recipient COS-7 cells. Phenotypic changes were evaluated by immunostain of the cells with the F-actin marker phalloidin. Cell were classified as “no change” (almost round cells without membrane protrusions) or as “change” (smaller somas with abundant protrusions of tiny filopodia and/or long membrane extensions) (Fig. 2F). Quantification of the cells displaying a phenotypic change showed that neuronal EV addition induced a significant increase in the number of “changed” cells in comparison with cells treated with COS-7-cell-derived EVs (lacking M6a) or with the culture media used for neuron maintenance or with conditioned media from primary neuron cultures (Fig. 2G). Even though conditioned media from neurons is expected to contain EVs, it is possible that their low concentration prevents large phenotypic changes as observed with isolated and concentrated EVs (see Discussion). RT-qPCR measurements in samples isolated from neuronal EVs showed undetectable *gpm6a* mRNA levels (Fig. S5), suggesting that the effects were not due to mRNA transferred from EVs to the recipient cells. Therefore, changes in the membrane morphology of recipient COS-7 cells could be attributed to proteins, including M6a, carried in the neuronal EVs.

**Figure 3:**
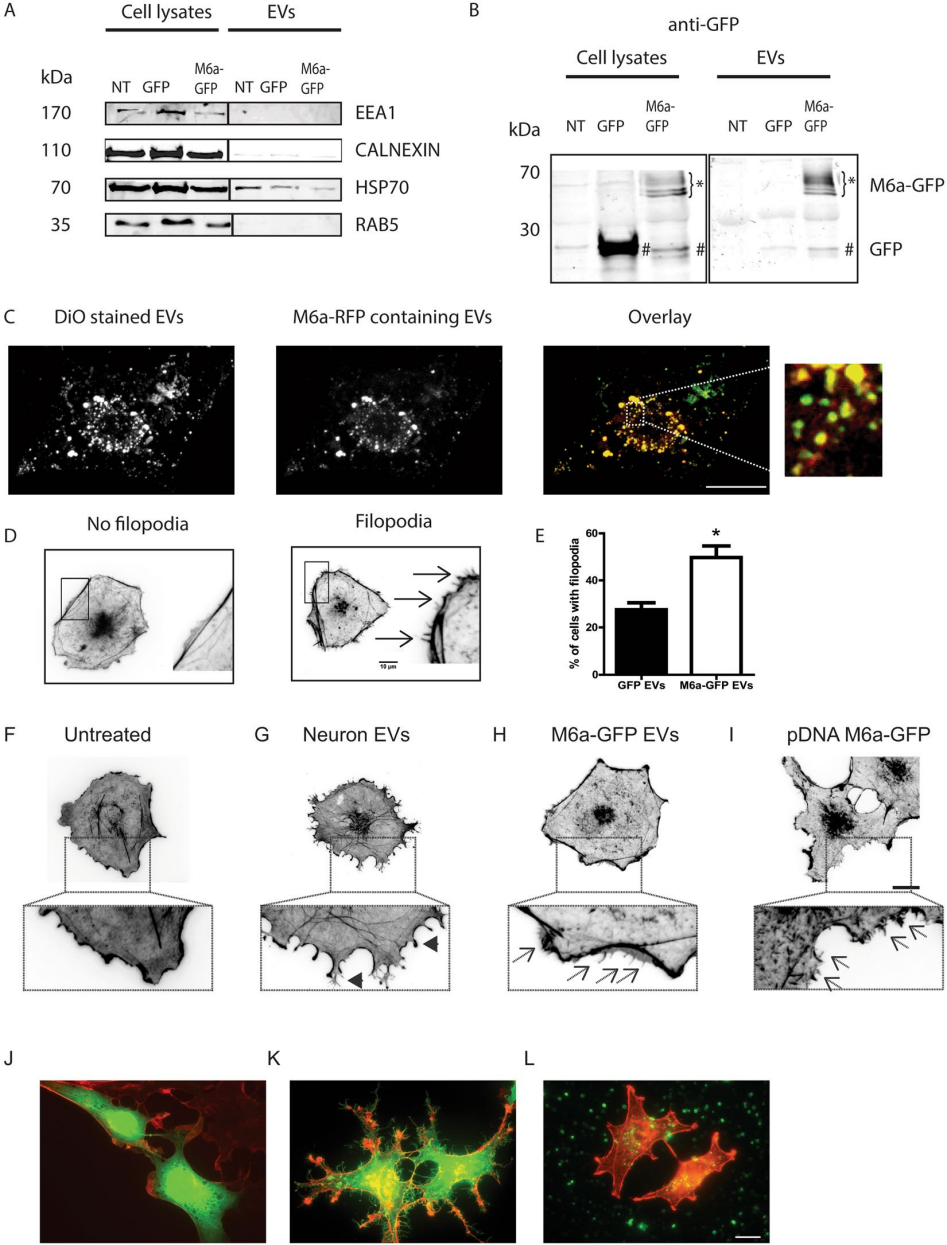
Phenotypic changes in COS-7 cells treated with M6a-containing EVs. EVs were isolated from COS-7 cells that were either non-transfected (NT), transfected with a plasmid coding GFP or with a construct coding the fusion M6a-GFP. (**A**) EV Western blot characterisation. Negative EV markers used were EEA1, calnexin and Rab5. HSP-70 was used as a positive EV marker. Similar amounts of protein (25 µg) were loaded in each lane. Western blot membrane was cut and incubated with the indicated antibodies. For comparison, cell lysates were included in the analysis. (**B)** The EV content assessed by Western blot with anti-GFP antibody to detect GFP and M6a-GFP. Bands representing M6a are indicated with stars while bands corresponding to GFP are indicated with hashtags. Full-length blots are shown in Figure S11. (**C**) Representative image showing EVs inside COS-7 cells. EVs were isolated from COS-7 cells transfected with M6a-RFP and subsequently stained with DiO lipophilic dye. Left panel shows the total population of EVs (DiO stain). Middle panel shows the EVs that carry the M6a-RFP protein. Right panel and magnifications show the overlay of left and middle images. Images show maximum projections of confocal Z-stacks. Scale bar 20 µm. (**D)** Phalloidin stained COS-7 cells were magnified to visualise filopodia. Recipient cells with phenotypic changes display multiple protrusions (arrows in right panel). (**E**) Quantification of the results reveals that M6a-positive EVs increased the percentage of COS-7 cells displaying filopodia compared to cells treated with EVs isolated from GFP-transfected COS-7 cells. n = 3 independent experiments, over 100 cells counted per each condition. *P < 0.05; t-student test. (**F**–**I)** Comparison of phenotypic changes between untreated COS-7 cells (**F**) or cells treated with neuronal EVs (**G**), M6a-GFP-containing EVs (**H**) and plasmid DNA coding M6a-GFP (**I**). Amplification of figures show no filopodia (**F**), long membrane extensions (arrowheads in **G**), long tiny filopodia in patches (arrows in **H**) or extensions throughout the cell surface (arrows in **I**). (**J–L**) Comparison of GFP expression between COS-7 cells transfected with a plasmid coding GFP **(J)**, the fusion protein M6a-GFP **(K)** and treated with EVs carrying the fusion protein M6a-GFP **(L)**. Cells were stained with phalloidin (red). Scale bar 20 µm.

The above experiments using neuronal EVs to induce changes in COS-7 cells do not allow to specifically attribute this effect to M6a. These EVs certainly carry other proteins in addition to M6a. To elucidate this issue, we analysed EVs isolated from COS-7 cell transfected with M6a fused to the green fluorescent protein (GFP). Transfection with GFP alone or untreated COS-7 cells were used as controls. After 3 days, EVs were isolated and reacted with the negative EV markers calnexin (ER), EEA1 and RAB5. HSP70 was used as a positive marker. Western blot analysis showed that all EV negative markers were present in cell lysates but none in the EV fractions (Fig. 3A). HSP70 were detected, as was expected, in all the samples, including the EV fraction. The marker profiles and the similar amount of protein loaded on each lane confirmed the identity and the enrichment of the EV fractions.

Taking into account that the only source of M6a in COS-7 transfected cells was the one fused to GFP, Western blot membranes were stained with anti-GFP antibody to reveal M6a presence. COS-7 cells transfected with GFP alone showed a reactive band at 27 kDa, the expected size of GFP (hashtags in Fig. 3B). The same reactive band was observed in cells transfected with the fusion M6a-GFP. In this lane, additional bands (60 kDa) corresponding to the fusion protein M6a-GFP were detected, indicating the heterologous M6a expression (stars in Fig. 3B). No reactive bands for M6a-GFP were detected in EVs from GFP- and non-transfected COS-7 cells.

Heterologous expression of M6a in COS-7 cells allowed us to obtain EVs carrying M6a, thus excluding most molecules from neural origin. These EVs carrying M6a were then used to study M6a participation in the phenotypic change induced in recipient COS-7 cells. EVs from COS-7 cells transfected with M6a fused to the red fluorescent protein (RFP) were isolated. This EV population was stained with the lipophilic dye DiO (green) and added to COS-7 cells. Figure 3C shows a representative confocal image of a COS-7 cell with incorporated EVs. All isolated EVs were stained with DiO (Fig. 3C left panel). However, due to an approximately 30% transfection efficiency, only a part of the total EVs contained the construct M6a-RFP (Fig. 3C middle panel). The EVs carrying M6a-RFP stained with DiO were observed in orange (Fig. 3C, right panel and magnification) indicating that heterologous M6a-RFP was effectively loaded to COS-7-cell EVs.

To study if M6a coupled to EVs retained the ability to induce membrane protrusions in recipient cells, vesicles from M6a-GFP- or GFP- transfected COS-7 cells were isolated and added to untreated COS-7 cells. Figure 3D shows representative images of COS-7 cells stained with the F-actin marker phalloidin and classified as cell with or without filopodia (arrows). This classification was the criteria used to quantify the effect of EV addition. Treatment of cells with EVs carrying M6a significantly increased the number of filopodium like protrusions in the membrane of COS-7 cells compared with cells treated with EVs from GFP-transfected cells (50% vs. 27.5%, *P < 0.05, Fig. 3E). These results show that M6a present in EVs can induce phenotype changes in recipient cells. Interestingly, these changes are different from those observed with EVs from neural origin (compare Figs 2E and 3D, see Discussion). Cells treated with neuronal EVs changed their form entirely (Fig. 3G). In contrast, in cells treated with M6a-containing EVs, filopodium induction was observed in discrete regions or patches (arrows in Fig. 3H). The M6a-containing EV effects on COS-7 cell surface also differed from the changes observed when COS-7 cells were transfected with a plasmid DNA encoding M6a-GFP (arrows in Fig. 3I), where filopodia are observed throughout cell surface. Notably, cells transfected with GFP (Fig. 3J) or M6a-GFP plasmid (Fig. 3K) expressed GFP, while cells treated with EVs carrying M6a-GFP (green dots in Fig. 3L) did not. These results indicate that phenotypic changes were induced by the M6a protein transferred to the recipient cells. PCR amplification of M6a from DNA isolated from EVs was negative (Fig. S6), indicating that plasmid DNA coding M6a used to transfect cells was not loaded in EVs. This result strengthens the notion that the phenotypic change did not occur through plasmid DNA transference but rather through protein transfer.

### Stress affects M6a glycoprotein levels in serum

**Figure 4:**
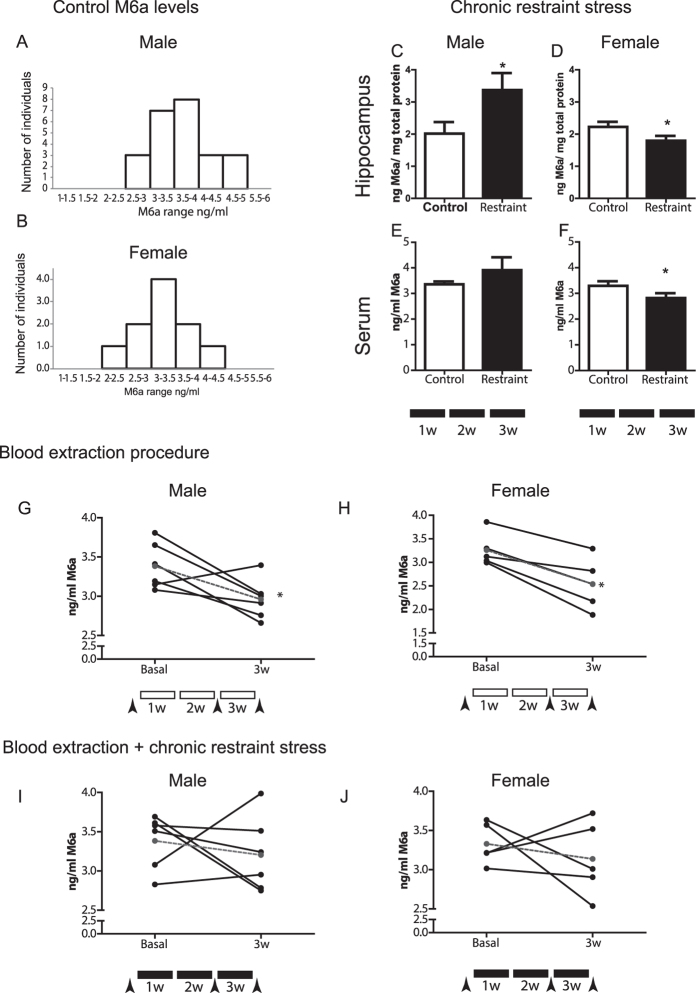
Peripheral M6a glycoprotein levels are affected by stress. (**A**,**B**) ELISA determination of M6a levels in serum samples of a control C57Bl/6 J mouse population. Both male (**A**) and female (**B**) samples show a normal distribution. Female mice n = 10; Shapiro-Wilk normality test *P* = 0.3; male mice n = 24; *P* = 0.6. (**C**-**F**) Female and male mice were subjected to a 3-week protocol of chronic restraint stress (CRS, black boxes). CRS consisted of 5 days of daily restraint stress followed by a 2-day interruption. Mice were sacrificed at the end of week 3. M6a levels in hippocampal homogenates (**C**, **D**) and in serum (**E**,**F**) were determined by ELISA. n = 9–10 for females and n = 6–8 for males. *P < 0.05. (**G**–**J**) To evaluate stress effect on M6a levels in individual male and female mice three blood extraction procedures were performed (arrowheads between boxes), the serum from the first and third extractions were assayed for M6a levels. (**G**, **H**) effect of blood extraction alone (white boxes) (**I**, **J**) blood extraction plus CRS (black boxes). Each line represents one individual. Gray line represents mean values. T-student test followed by Dunnet multiple comparison test *P < 0.05. n = 6 for males and n = 5 for females.

Brain M6a mRNA levels are affected by stress. Then, the M6a presence in blood appears as a possible tool to assess stress effects in peripheral fluids, such as serum. Therefore, we first studied differences in M6a levels among individual animals in serum samples, and found that data showed a Gaussian distribution when plotted as frequency of M6a levels for both male and female control mouse cohorts (Fig. 4A, B; Shapiro-Wilk normality test for female mice *P* = 0.2 and male mice *P* = 0.9). Moreover, M6a level medians did not differ between genders (male median 3.71 ng/ml, female median 3.41 ng/ml; Mann-Whitney test *P* = 0.1168). This result shows that in a natural population, M6a levels somewhat differ between individuals and allowed us to establish that M6a serum levels ranged from 2 to 4.5 ng/ml in females and from 2.5 to 5 ng/ml in males.

Previous studies from our group indicated that chronic stress affects M6a mRNA levels particularly in the hippocampus^18, 19^. Thus, we tested whether the glycoprotein M6a levels in hippocampus and in serum vary under stressful conditions using an interrupted restraint protocol. Chronic restraint stress (CRS) resulted in a decrease in body weight gain compared with non-stressed mice (Fig. S7A,B). In addition, chronic stress reduced the number of oestrous cycles in females along the 3 weeks of CRS (Fig. S7C). Both alterations indicate that animals were stressed^30^. CRS (black boxes in Fig. 4C–F) significantly increased in M6a levels in male hippocampus (Fig. 4C). Conversely, a significant decrease of M6a glycoprotein was observed in female hippocampus (Fig. 4D). Interestingly, a similar tendency, decrease and increase M6a levels in female and males, respectively, was observed in serum samples of CRS mice compared with control animals (Fig. 4E,F). Since CRS induces different changes in the hippocampus, including changes in M6a expression, the measurement of serum M6a levels might be used to monitor stress in peripheral fluids.

These results encouraged us to evaluate serum M6a levels in a follow-up experiment, where each individual animal was its own control. To this, a blood extraction schema (arrowheads and white boxes in Fig. 4G,H) was added to the CRS protocol (arrowheads and black boxes in Fig. 4I,J). The blood extraction procedure itself affected M6a levels in serum in both male and female animals. After the last blood extraction, most mice (individually analysed, black lines in Fig. 4G,H) showed a reduction in serum M6a in comparison with their own M6a levels at the beginning of the experiment (basal vs. 3w, Fig. 4G,H). Significant differences were found for mean values (dashed lines, *P < 0.05).

When mice were exposed to CRS and, in addition, subjected to the blood extraction procedure, we observed a large variation among individuals. Serum M6a levels either increased, decreased or remained invariable in the different animals (compare basal vs. 3w, Fig. 4I,J). Furthermore, the extent of this variation depended on each individual. Sample size limitations when working with mice preclude further conclusions that require a large follow-up in individuals to analyse daily fluctuations among other variables (see Discussion). Altogether, these results show that M6a levels in blood vary under different stressful conditions.

## Discussion

Glycoprotein M6a participates in neurite outgrowth, filopodium formation^1, 4^ and in synaptogenesis^2, 3^. Different animal models showed that M6a mRNA levels are modulated by chronic stress in the brain^17–19^. In this work, we investigated an unexplored area: the detection of the glycoprotein M6a in peripheral fluids such as serum. It has been already depicted the M6a presence in human urine and in cerebrospinal fluid^5–7^. Now, our findings demonstrate the presence of this protein in blood.

M6a is an integral membrane protein and, consequently, it was not expected to be soluble in blood. Therefore, taking into account that EVs are enriched in glycoproteins and in transmembrane proteins^31, 32^, we studied the possibility that M6a is transported coupled to EVs. In support of this idea, M6a was previously identified in large scale proteomic analysis of EVs from urine and from mesenchymal stem cells^5, 33^. EVs were isolated from serum by differential ultracentrifugation, obtaining a heterogeneous EV population, some EVs carried the tetraspanin CD63 (a well-known EV marker), some carried M6a and others, both. EVs are enriched in glycoproteins, tetraspanins and proteins that associate with lipid rafts^27, 34^. Interestingly, M6a shares some tetraspanin structural features and associates with cholesterol-rich domains^35^. Since some EVs were labelled by M6a, we propose it as a possible EV marker. M6a is highly expressed in the brain where its main functions have been observed^1, 23, 36, 37^. Hence, M6a presence in EVs isolated from serum suggest that, at least in part, serum M6a could be originated in the brain. Indeed, approximately 8% of blood EVs derive from nervous system^38^. Moreover, EVs can pass the blood-brain barrier^39^ reinforcing the possibility of a neural origin for serum M6a.

As mentioned, neuronal EVs modify the recipient cell phenotype. Since neuronal EVs contain several cargoes that could promote membrane protrusion formation in recipient cells besides M6a, we overexpressed M6a fused to GFP in the non-neuronal COS-7 cell line to produce EVs carrying the fusion protein. These EVs carry M6a but lack most of the other neuronal proteins. Recipient COS-7 cells treated with M6a-loaded EVs showed phenotypic changes restricted to filopodium induction, precisely, the activity described for M6a. Furthermore, the difference between the phenotypic changes induced by plasmid DNA transfection or neuronal EVs (filopodia throughout cell surface) and by M6a-containig EVs (filopodia in patches) suggests that EVs may transfer M6a protein that may be locally inserted in the plasma membrane to induce filopodium formation. We cannot, however, discard that M6a overexpression in COS-7 cells induces the expression of additional molecules that could be also incorporated into vesicles.

EVs are involved in intercellular communications via the transfer of proteins, lipids and nucleic acids^40^. EVs specifically target recipient cells and exchange proteins triggering downstream signalling^41^. Our findings show that M6a-containing EVs derived from hippocampal neurons stimulate membrane protrusion formation in COS-7 cells. Kanada *et al*.^42^ have proposed that the molecule transferred to produce the phenotypic change induced by EVs is usually plasmid DNA carried in the vesicles^42^. In our studies, the induction of the filopodial phenotype seems to be achieved by horizontal protein transfer. No green recipient cells were observed upon EV transfer. If DNA or mRNA were responsible for the phenotype change, it would have been expected that expression of the fusion protein results in green recipient cells. Furthermore, no M6a DNA or mRNA were detectable in M6a-containing EVs. Horizontal protein transfer has been described in a limited number of cases. Among them is the chemokine receptor CCR5, the principal co-receptor for macrophage-tropic human immunodeficiency virus 1. Microparticles containing CCR5 can transfer the receptor to non-expressing-CCR5 cells and render them CCR5 positive enabling virus infection^43^. EV protein transfer has been proposed as one way for the propagation of Prion proteins from the periphery to the CNS^15, 44^.

M6a-GFP, but not GFP alone, was detected in the EVs from cells transfected with M6a-GFP or GFP, respectively. This result supports the idea that EV upload is not random. Accordingly, it has been demonstrated that soluble proteins have to be coupled to ubiquitine to be sorted to EVs^45^. As mentioned before, EVs are enriched in tetraspanins and proteins that associate with lipid rafts^27, 34^, which favour upload into EVs of proteins that oligomerise. Interestingly, M6a associates with lipid rafts^35^ and dimerises through its transmembrane domains^4^. Thus, our results suggest that EVs can be selectively loaded with heterologous proteins (e.g. M6a) to transfer information from one cell to others. It remains to be identified which are the target cells where M6a-containing EVs can affect cellular plasticity. Neuronal EVs containing glutamate receptor (GluR2) subunits are thought to participate in synaptic plasticity^11^. EV release is regulated by synaptic activity^46^. In addition, in the central nervous system, EVs can be released and endocytosed/phagocytosed by glial cells, including astrocytes, modulating their role at synapses^44^. Similarly, M6a-containing EVs may stimulate cell-cell communication and plasticity at different subcellular structures, including synapses.

Since chronic stress modulates the M6a mRNA levels in the brain, the M6a presence in serum opened the opportunity to analyse the possible correlation between stress and M6a protein levels in peripheral fluids. Although we cannot indicate the actual origin of the M6a found in serum nor its role in the response to stress, our results show that the glycoprotein M6a in serum associated with an EV subset and that stress altered M6a levels. M6a serum levels were analysed in a control (naive) cohort of male and female C57Bl/6 mice showing for both genders a normal distribution. Chronic stress affected serum M6a levels in a sex specific manner. While females showed a reduction in M6a levels both in serum and hippocampus, males exhibited the opposite pattern﻿. At first, this seems to contradict previous results from our group^17, 18^ but it has to be taken into account that in the present work an interrupted restraint protocol was used and we focused on M6a protein and not on mRNA levels. The differences in the stress response seen here between males and females suggest that there is a gender-specific response to stress regarding to M6a levels. Interestingly, gender differences have been observed in the hippocampus morphology of animals exposed to chronic stress. While males show apical dendritic atrophy of pyramidal neurons, females do not and show a decrease in the number of branch points in the basal dendritic tree^47^. Since M6a participates in the establishment of neuronal morphology, the gender differences in M6a levels may be partially responsible of the distinctive morphological changes.

M6a response in blood was also dependent on the stress protocols applied to mice. Repeated blood extraction is known to be a stressful situation *per se* as shown before^48, 49^. Addition of this stressful situation (blood extraction) to the chronic restraint stress protocol resulted in a variable response among animals. We hypothesise that blood extraction, acting as a stress inoculation, gave the animals an opportunity to learn and improve coping strategies. Similarly, Mc Ewen *et al*. (2015) reported that the effects of stress on each individual depend on the history of stress exposure and this impacts on recovery and adaptation to stress^50^. Future research will be directed to study if the individual traits seen in the stress response can be related to M6a levels resulting in an alteration of spine reorganisation or dendritic arborisation.

Altogether the CRS and the follow-up experiment demonstrated that serum M6a levels are sensitive to different type of stress. In the central nervous system, all cell types have been shown to release EVs^51^ that can either be taken up by neighbouring cells or released into cerebrospinal fluid and blood. Changes in serum M6a levels may reflect the final balance of M6a levels in all areas of the brain. Because of the M6a activity, we hypothesise that M6a in the EVs may serve as a positive signal to promote neuronal plasticity and to counteract the deleterious effects of stress. We propose M6a as an interesting candidate that can be detected in body fluids to be further studied in the field of stressful events. In this sense, preliminary results and ongoing studies suggest that M6a can also be detected in saliva, an alternative that will certainly simplify sample extraction and patient follow-up studies.

## Methods

### Animals

All experiments performed with animals in this study were approved by the Committee on the Ethics of Animal Experiments of the Universidad Nacional de San Martin (CICUAE-UNSAM #004/2015) and were carried out in accordance with the recommendations of the Guide for the Care and Use of Laboratory Animals of the National Institutes of Health and the guidelines laid down by the Committee for the Care and Use of Animals for Experimentation. Mice were obtained from The Jackson Laboratory and bred in our facilities. Female and male mice C57BL/6 J (60 to 90 days old) were used in all experiments.

### Serum obtaining

To obtain blood, orbital sinus or tail vein extraction procedures were used. Blood samples were incubated at 37 °C to allow clot formation, then they were centrifuged 5 min at 5000 rpm to obtain serum samples.

### Cerebrospinal fluid obtaining

Mice were anaesthetised and the cerebrospinal fluid (CSF) was obtained from the Cisterna Magna by puncture as described by Liu & Duff (2008)^52^.

### Protein quantification, Western blots and antibodies

Protein content was quantified using the Bradford dye-binding method (Bio-Rad Laboratories). Primary antibodies used: polyclonal rabbit antibody against C-terminus of M6a (1/1000) developed in our laboratory^24, 35^, polyclonal rabbit antibody against C-terminus of M6a (Aviva Systems Biology, 1/500) monoclonal mouse antibody against Flotillin-1 (BD Bioscience, 1/1000); polyclonal rabbit antibody against GFP (Invitrogen, 1/1000); polyclonal rabbit antibody against Rab5 (Cell Signaling, 1/500); monoclonal mouse antibody against EEA1 (BD Bioscience, 1/1000); Calnexin (Sigma, 1/2000) and Hsc70 (Affinity BioReagent, 1/1000). Blot images were processed to show each band more clearly. In all cases, processing was applied equally across the entire image. In some cases, individual lanes were cropped. Full -length blots are shown in Supplementary Figures S8–S12.

### EV isolation

EVs from COS-7 cell, primary neuron culture medium or from serum pool were prepared by ultracentrifugation as described earlier^26^ with minor modifications. See Fig. S2. For small volume samples, to concentrate EVs or after labelling EVs with DiO, a Polyethylene glycol (PEG) based method was used as described by Rider *et al*.^53^. See also Supplementary Information for further details. All the isolated EVs were stored at −80 °C until further use.

### Transmission electron microscopy (TEM)

TEM was performed at the LANAIS-MiE-IBCN (SNM-CONICET) facility (Buenos Aires, Argentina). Sample preparation was done according to Théry, 2006^26^. Micrographs were acquired in a Carl Zeiss EM 109 T transmission electron microscope operating at 80 kV and equipped with a Gatan ES1000W (11 Mpixel) digital camera.

For immunogold stain M6a antibody (MBL International, Woburn, MA) was used and complexes were detected using 18 nm gold conjugated anti-rat IgG antibody (Jackson ImmunoResearch, WestGrove, PA). Negative stain and acquisition was performed as described before. (See Supplementary Information).

### EV stain and immunostain

Isolated EVs were stained with the lipophilic dye DiO (Invitrogen V22886) 1/200 in PBS at 37 °C for 1 h. For stain removal, PEG purification was carried out. Direct immunostain of EVs was performed according to Athman *et al*.^54^. Briefly, EVs were fixed with formaldehyde and spotted onto coverslips. Coverslips were then blocked with 10% bovine serum albumin in PBS. Next, coverslips were incubated anti-CD63 (1/10; Developmental Studies Hybridoma Bank) or anti-M6a (1/200; MBL International), incubated with secondary antibodies and finally mounted with FluorSave™ reagent (Calbiochem, San Diego, CA, USA)﻿.

### Hippocampal cultures

Dissociated neuronal cultures were prepared from hippocampi of embryonic day 19 Sprague–Dawley rats obtained from the Faculty of Veterinary Sciences (Buenos Aires, Argentina), as described by Brocco *et al*.^55^. For further details, see Supplementary Information. Hippocampal neurons were cultured for 14 days and conditioned medium was collected for EV isolation.

### Cell line and transfection

COS-7 cells purchased from ATCC (Manassas, VA) were transfected with polyethylenimine (PEI; Faculty of Pharmacy and Biochemistry, University of Buenos Aires) following the manufacturer’s instructions. The plasmids used were the pEGFP-C1 or pRFP-C1 (Clontech) encoding the enhanced green fluorescent protein or the red fluorescent protein respectively and the same expression plasmids fused to M6a.

### Immunostain and filopodium quantification

COS-7 cells (8 × 10^3^) were treated with the EV solution (50 µg protein/well in a 24-multiwell plate format) for 72 h. These conditions were standardised in previous dose-response experiments (Fig. S3). Then, cells were fixed and F-actin was stained with rhodamine red- or Alexa Fluor 488-conjugated phalloidin (1/1000; Molecular Probes). Alternatively, plasma membranes were visualised by transfection with a plasmid that encodes a PI(4,5)P2 lipid selective PH domain from phospolipase C (PLC) fused to RFP fluorescent protein that targets cell membrane. Coverslips were mounted in FluorSave™ Reagent. Fluorescent images were acquired using Nikon E600 microscope with epifluorescence illumination (Plan APO 1009 oil, 1.4 NA objective) or Olympus FluoviewFV1000 confocal laser scanning microscope (Plan APO N 609 oil, 1.42 NA, FN 26.5 objective) with FV10-ASW software. Images were processed with the ImageJ software. The Orthogonal Views tool was used to show EV localisation. The percentage of cells showing membrane protrusions was calculated in 100 cells per condition from 3–4 experiments. All experiments were blind scored.

### Stressors

For the end-point experiment (Fig. 4C–E), naive mice were stressed chronically for three weeks. Mice were individually placed into transparent plastic tubes fitted closely to their body for 4 h (10AM-14PM) per day, five days /week followed by a two-day break with no stress. Interrupted restraint protocol was based on the one described by Zhang *et al*.^56^ as it helps avoid mice habituation to the stress protocol^56^. Naive age-matched unstressed animals were used as controls. At the end of the stress protocol, blood was collected from the orbital sinus. After that, animals were sacrificed and brains were removed for hippocampus isolation. Hippocampal tissue was homogenised in phosphate buffered saline (PBS, 1 mg tissue/100 µl). Total protein was quantified by Bradford dye-binding method, 2–2.5 µg/µl were obtained.

In the follow-up experiment (Fig. 4E–H), naive mice were subjected to a first blood extraction procedure (tail vein extraction). Then the animals were randomly assigned to control or chronic restraint stress (CRS) group. The CRS animals were subjected to the same interrupted restraint protocol used for the end-point experiment. At the end of the second week, blood from animals of both groups was collected by tail vein extraction. At the end of the protocol (third week) blood was collected from the orbital sinus.

For both protocols, serum was obtained from all blood samples and analysed by ELISA. Both serum and tissue samples were processed according to ELISA kit #MBS9325454 MyBioSource (San Diego, CA, USA) following manufacturer’s instructions. An n of 13–15 animals per group was used for the restraint experiment. An *n* of 5–8 animals per group was used for blood extraction/blood extraction plus restraint stress.

### Statistical analysis

Statistical analysis and graphs were carried out with GraphPad Prism Version 5.00.288. All data were subjected to normality and equal variance testing using IS version 2010 (Infostat software, Grupo InfoStat, FCA, Universidad Nacional de Córdoba, Argentina). Results were reported as mean ± SD. When two groups were compared the t-test or its non-parametric version Mann-Whitney U-test were used. One-way analysis of variance (ANOVA) test followed by Bonferroni post test was used to compare more than 2 groups. In follow-up experiments (Female mice n = 5; male mice n = 8 per group**)**, one-way ANOVA followed by Dunn’s test to compare each treatment to the control group (basal) was used. For all tests, a *P* < 0.05 was considered statistically significant.

## Electronic supplementary material

Supplementary File
