# Supplementary material for: Neural glycoprotein M6a is released in extracellular vesicles and modulated by chronic stressors in blood
Source: Sci Rep. 2017 Aug 29;7:9788. doi: 10.1038/s41598-017-09713-0 (PMC5575271; doi:10.1038/s41598-017-09713-0)
Supplement: Supplementary file 1 — Supplementary File [file 41598_2017_9713_MOESM1_ESM.pdf]

Neural glycoprotein M6a is released in extracellular vesicles and modulated by chronic stressors in blood

Melisa C. Monteleone[1], Silvia C. Billi[1], Marcela A. Brocco[1], Alberto C. Frasch[1]

1 Instituto de Investigaciones Biotecnológicas - Instituto Tecnológico de Chascomús (IIB-INTECH). Universidad Nacional de San Martín - Consejo Nacional de Investigaciones Científicas y Técnicas (UNSAM-CONICET). Av. 25 de Mayo y Francia. CP: 1650. San Martín. Buenos Aires. Argentina.

Corresponding author: Marcela A. Brocco

SUPPLEMENTARY INFORMATION

Stress assessment. Animals were weighed twice per week. Body weight gain was determined as final weight relative to initial body weight (Fig.S2A and B). Estrus synchronization in female mice was achieved by keeping the mice in a cage that contained soiled bedding from four reproductively mature male for two days before experiment onset. To ensure that females were cycling, vaginal smears were daily analyzed for estrous cycle stage determination (Fig. S2C).

Cell lines and transfection. COS-7 cells were cultured in DMEM supplemented with 10% (vol/vol) FBS, penicillin, and streptomycin. For EVs isolation, the FBS was depleted of vesicles by ultracentrifugation and the supernatant was filter sterilized (0.22 μm) before using for cell culture. For filopodia quantification, 20% depleted-vesicle FBS was used to avoid COS-7 differentiation.

EVs isolation. Conditioned medium from COS-7 cells and primary neurons was collected and sequentially centrifuged (15 min at 2,000xg at 4°C and 30 min at 20,000xg at 4°C). The final supernatant was ultra centrifuged at 100,000xg to pellet the EVs. The resulting pellet was resuspended in PBS for Western Blotting, electron microscopy, immunocytochemistry or for cell treatment (Fig. S3).

Hippocampal cultures. Hippocampi from fetuses were dissected and pooled to obtain a single-cell suspension. Briefly, tissue was treated with 0.25 % trypsin in Hank's solution for 15 min at 37 °C. A single cell suspension was prepared by dissociation in 10 % horse serum (Sigma) supplemented Neurobasal (Gibco) with 4.5 g/l glucose, 2 mM glutamine (Sigma), 100 U/ml penicillin and 100 μg/ml streptomycin (Sigma). Cells were seeded at a density of 60,000 cells/cm^2 on coverslips or 35 mm glass bottom-Petri dishes, coated with 0.1 mg/ml poly-L-lysine hydrobromide (Sigma, St. Louis, MO) and 20 μg/μl laminin (Gibco, Carlsbad, CA). After 2 hs, medium was changed to a serum-free medium, i.e. Neurobasal with 2 mM glutamine, 4.5 g/liter ovalbumin (Sigma) and B27 serum-free supplements (Gibco).

PEG-based EVs isolation method. A 50% PEG solution (average molecular weight of 8,000, Sigma) was added to the vesicle-containing medium. The latter was centrifuged for 15 min at 2000xg at 4°C and then for 30 min at 20, 000xg at 4°C. 200 µl of PEG solution was used per ml of extracellular fluid. After PEG addition, samples were mixed thoroughly by inversion and incubated overnight (at least 12 hrs) at 4 °C. The next day, samples were centrifuged at 20,000xg and the resulting pellet was resuspended in 50–500 µL of PBS (pH 7.4). All the isolated EVs were stored at – 80 °C until further use. Freeze and thaw cycles were avoided.

Blocking peptide assay. To confirm the presence of M6a, a synthetic peptide able to block commercial anti-M6a (Aviva Systems Biology), was used. 4.7 µg of blocking peptide was incubated with 1 µg of the M6a antibody overnight at 4°C. Antibody with no blocking peptide was incubated in the same conditions.

BSA depletion. For serum M6a detection, removal of albumin was performed according to Chen *et al.* (2005) (1). The resulting pellet was resuspended in rehydration buffer (8M Urea, 5% beta-mercaptoethanol and 2% SDS).

Immunogold stain. For immunogold stain the grid was washed in 0.05 M glycine/PBS and blocked using 1% BSA/PBS. The grid was washed and transferred to a 1:25 dilution of the anti-M6a antibody (MBL International, Woburn, MA) for 45 minutes at room temperature. The grid was then incubated with a 18 nm gold conjugated anti-rat IgG antibody (Jackson ImmunoResearch, WestGrove, PA) for 60 minutes at room temperature. The grid was washed again and incubated with 1% glutaraldehyde for 5 minutes. Negative stain and acquisition of images was performed as described before.

Direct EV immunostain. The method for EV immunostain was extracted from Athman et al 2015 (2). Briefly, frozen stocks of purified EVs were thawed and fixed in a final concentration of 2% electron microscopy grade formaldehyde, immediately spotted onto coverslips and allowed to adhere for 20 min in a humidified chamber. Coverslips were then incubated for 20 min with 150 µl of 2% electron microscopy grade formaldehyde,

washed three times with 150 µl PBS, and blocked for 30 min with 10% BSA in PBS. Two sequential labeling steps were performed. First, coverslips were incubated for 30 min with 5 µg/ml anti-CD63 (Hybridoma Bank) or anti-M6A (MBL International), washed three times with 150 µl PBS, incubated for 30 min with 5 µg/ml Alexa Fluor 568 or 488 anti-mouse IgG or with 5 µg/ml TRITC anti-rat IgG , washed three times with PBS and mounted on microscope slides containing 7 µl FluorSave reagent.

PCR. M6a complete coding sequence was amplified using Taq polymerase (Invitrogen). As template plasmid DNA (pDNA) or EVs isolated from COS-7 transfected cells were used. To rule out the presence of PCR inhibitors, a pDNA spike was added to the EVs. Primers used were: Fwd 5'GCC ACC GGT ACC ATG GAA GAG AAT ATG GAG A3'; Rev 5'GGC CCA AGC TTT TAT GTG TAT GCA TTG AGC CG3'

RT-qPCR. To isolate EVs, 5 x 10^6 neuronal cells were cultivated for 14 days and EVs were isolated as described before from 8 ml of culture media. Then EVs were homogenized in TRIzol® Reagent (Life Technologies, Rockville, NY, USA). Total RNA was isolated with DirectZol RNA Miniprep (Zymo Research, Irvine, CA, USA) following manufacturer's instructions. All RNA obtained was used to synthesize complementary DNA by retrotranscription using oligodT and SuperScriptTM II Reverse Transcriptase enzyme (Life Technologies) according to the manufacturer's instructions. qPCR reactions were achieved with Kapa SYBR fast qPCR kit (KAPA Biosystems, Woburn, USA) using 4 µl diluted (1/2) cDNA. All qPCRs were carried out in a 7500 Real-Time PCR System (Applied Biosystems, Foster City, California, USA) as described previously (3). Primer sequences were designed using Primer Express 3.0 software (Applied Biosystems). Primer sequences for genes in table 1 in S6 are: *Cyclophilin-a* (NM_022536.2) F 5'-AAGCATACAGGTCCTGGCATCT-3' R 5'-CATTCAGTCTTGGCAGTGGCAG-3'; *Ywhaz*: tyrosine 3-monooxygenase/tryptophan5-monooxygenase activation protein, zeta polypeptide (NM_013011.3) F 5'-GATGAAGCCATTGCTGAACTTG-3' R 5'-GTCTCCTTGGGTATCCGATGTC-3'; *Gpm6a*: Glycoprotein M6A (NM_178105.2) F 5'-GCAGAAGTATGAAGACATCAAGTCAAA-3' R 5'-GTATGCATTGAGCCGCTCTTT-3'; *RpL13a* ribosomal protein L13a (NM_009438.5) F 5'-

TCCTCCAGAGTGGCTGTCCACT -3’ R 5’- AGCCTACCAGAAAGTTTGCTTACC-3; *Gapdh* (NM_001289726.1) F 5’-ACCACAGTCCATGCCATCAC-3’ R 5’- TCCACCACCTGTTGCTGTA-3. *Cyclophilin-a, Ywhaz, Rpl13a* and *Gapdh* mRNA has been previously detected in EVs (4–7). Moreover, some are usually used as housekeeping genes. Therefore we selected them as positive controls for this experiment.

1. Chen YY, et al. (2005) A modified protein precipitation procedure for efficient removal of albumin from serum. *Electrophoresis* 26(11):2117–2127.
2. Athman J, et al. (2015) Bacterial membrane vesicles mediate the release of Mycobacterium tuberculosis lipoglycans and lipoproteins from infected macrophages. *J Immunol* 195(3):1044–1053.
3. Fernández M, Alfonso J, Brocco M, Frasch A (2010) Conserved cellular function and stress-mediated regulation among members of the proteolipid protein family. *J Neurosci Res* 88(6):1298–308.
4. Lotvall J, Valadi H (2007) Cell to cell signalling via exosomes through esRNA. *Cell Adh Migr* 1(3):156–158.
5. Spanu S, Van Roeyen CRC, Denecke B, Floege J, Mühlfeld AS (2014) Urinary exosomes: A novel means to non-invasively assess changes in renal gene and protein expression. *PLoS One* 9(10). doi:10.1371/journal.pone.0109631.
6. Chiba M, Kimura M, Asari S (2012) Exosomes secreted from human colorectal cancer cell lines contain mRNAs, microRNAs and natural antisense RNAs, that can transfer into the human hepatoma HepG2 and lung cancer A549 cell lines. *Oncol Rep* 28(5):1551–1558.
7. Yang J, Wei F, Schafer C, Wong DTW (2014) Detection of tumor cell-specific mRNA and protein in exosome-like microvesicles from blood and saliva. *PLoS One* 9(11). doi:10.1371/journal.pone.0110641.

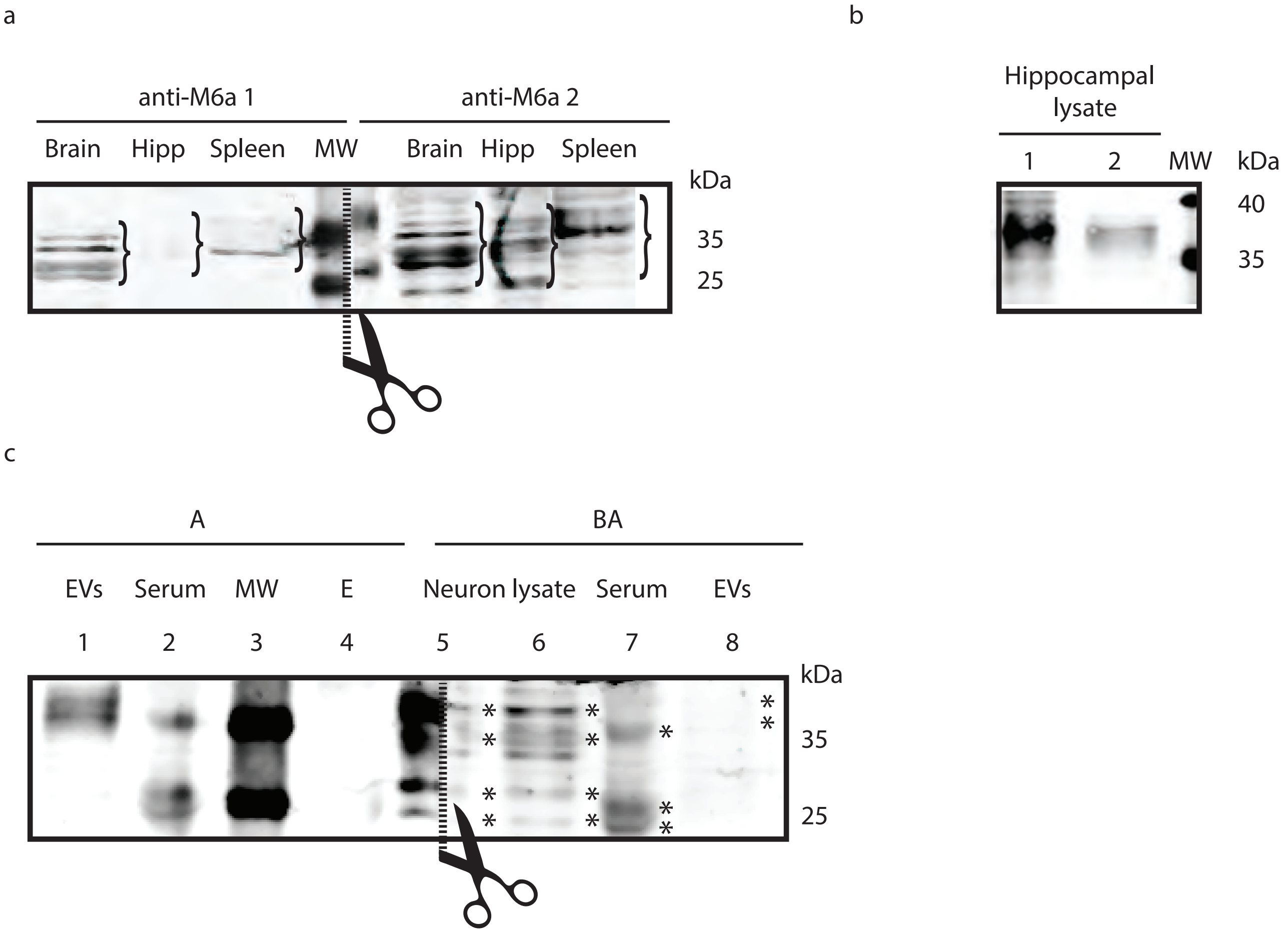

Figure S1. M6a has a different migration pattern according to tissue origin, antibody used for detection and extraction buffer used to isolate proteins. a) Western blot shows differential migration pattern and also different band intensities according to the tissue from where it was extracted. Moreover, the pattern is also dependent on the antibody used. In this case, left side of the membrane was incubated with antibody 1 (comercial) and the right side with antibody 2 (home-made), both raised againts M6a C-terminal region. Scissors represent a cut in the blotting membrane. b) Buffers used for protein extraction also have an impact on M6a migration pattern and bands intensities. In lane 1 and 2 the same ammount of protein was loaded. In lane 1 UREA buffer was used while in lane 2 Laemmli sample buffer followed by boiling. c) Western blot with comercial anti-M6a antibody blocked with a specific peptide. Scissors represent a cut in the blotting membrane. Left side was incubated with anti-M6a alone (A); right side of the membrane was incubated in the presence of blocked antibody (BA). Note that the blocking peptide severely reduces M6a signal indicated with stars in all sources analyzed. E: empty lane; EVs: extracellular vesicles from neuron culture; hipp: hippocampus lysate; MW: molecular weight marker. Uncropped images of full-lenght blots are shown in Figure S10, at the end of this file.

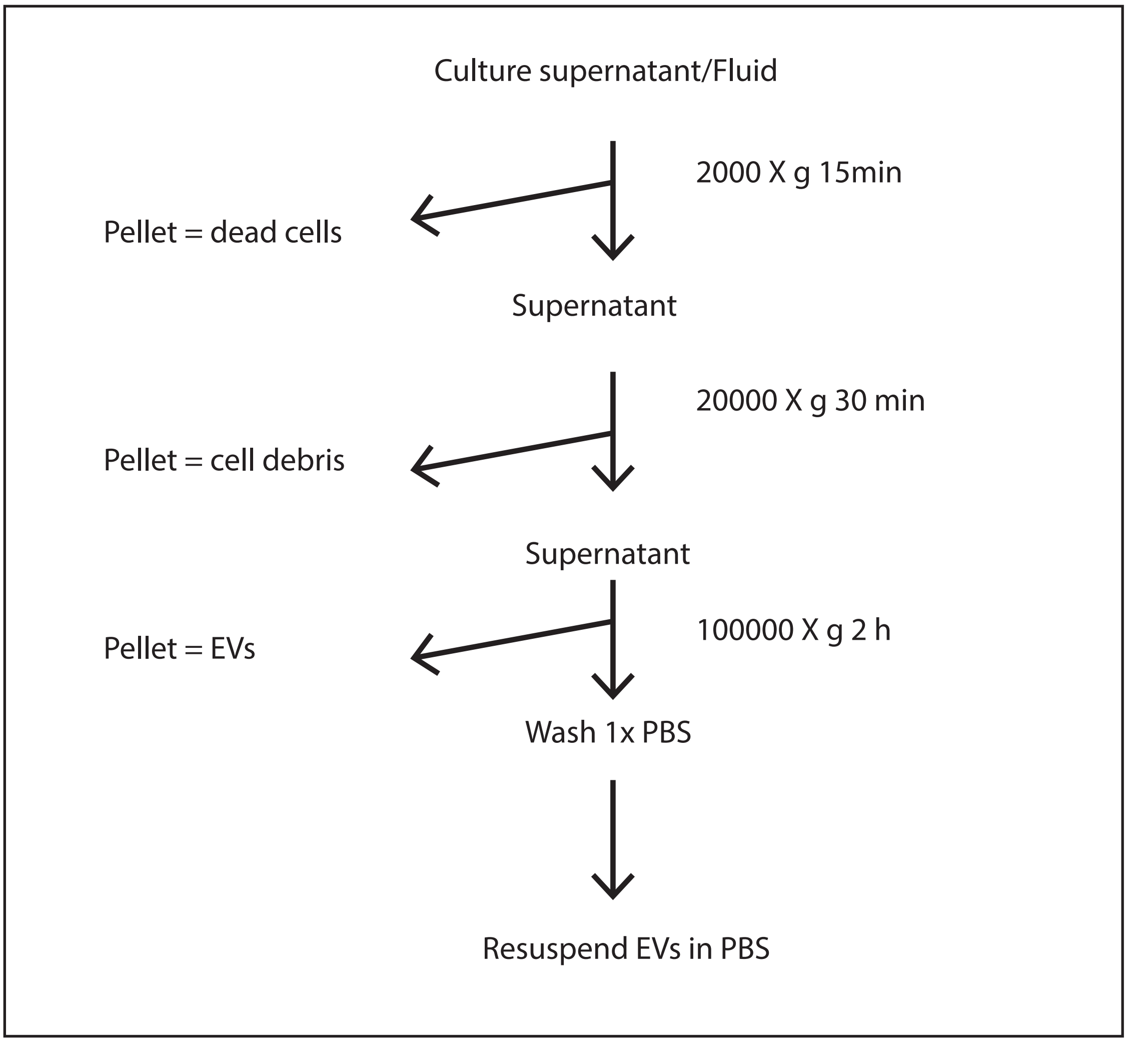

Figure S2. Flow chart for the EV purification procedure based on differential centrifugation. The speed and length of each centrifugation are indicated to the right of the chart. After each of the first two centrifugations, pellets (cells, dead cells, cell debris) are discarded, and the supernatant is kept for the next step. In contrast, after the 100,000×g centrifugation and PBS wash, pellets (EVs) are kept, and supernatants are discarded. Modified from Thery 2006.

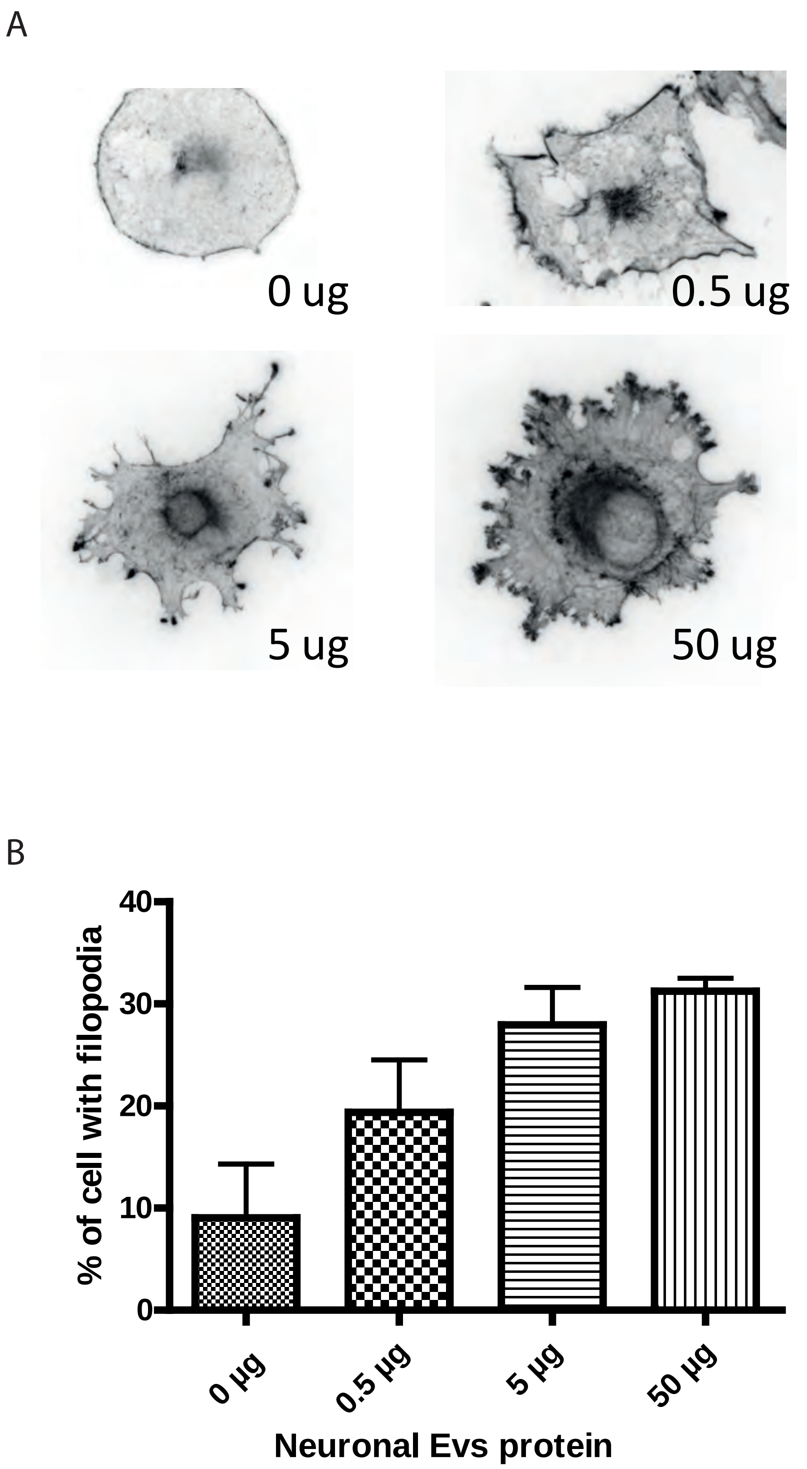

Figure S3. Dose-response curve for EV treatment of COS-7 cells. EV from culture medium of primary hippocampal neurons were isolated by ultracentrifugation and protein content was quantified. (A) COS-7 cells (6500-8000/well) were treated with 0 (n = 61 cells), 0.5 (n = 296 cells), 5 (n = 374 cells) and 50 (n = 503 cells) µg exosome protein/well. (B) Quantification of the number of cells exhibiting filopodia .

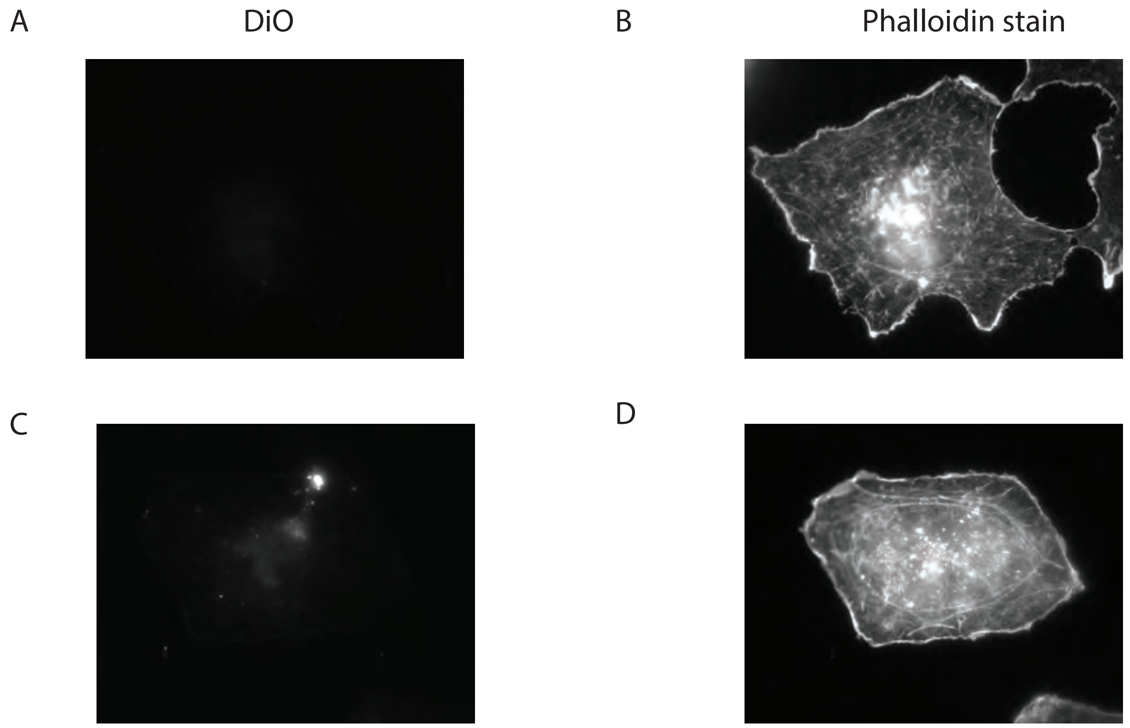

Figure S4. Fluorescence intensity is not due to residual dye in the supernatant during DiO-labeling of EVs nor due to PEG precipitaded free dye. A) COS-7 cells incubated with the supernantant from EVs stained with DiO (green channel). C) COS-7 cells treated with PEG pellet of fresh Neurobasal medium without (EVs), pre incubated with DiO. B,D) The same cells also were stained with phalloidin (red channel).

Target ↓	Ct →	**EVs from neuron culture**	NTC (**N**o **T**emplate **C**ontrol)	NRTC (**N**o **R**etro**T**ranscriptase **C**ontrol)
gpm6a		**34.8**	ND	**35**
ywhaz		29.7	39	ND
cyclophilin-a		28	32	32
rpl13a		27	33	33
gapdh		27	32	33

Figure S5: Neuronal EVs RT-qPCR measurements of *gpm6a* mRNA levels as well as of previously reported mRNAs present in EVs: *ywhaz*; *cyclophilin-a*; *rpl13a* and *gapdh*. Measurements are shown as Ct values (Threshold cycle). Note that Ct values for all mRNAs, except for *gpm6a* differ in, at least, 4 Cts with both negative controls.

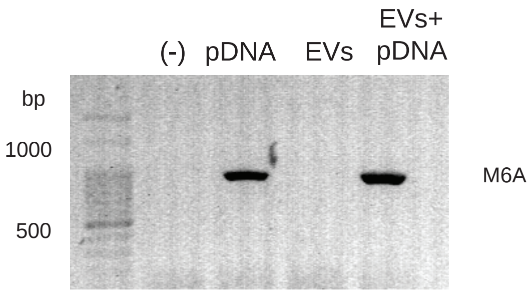

Figure S6. PCR fails to detect plasmid DNA on EVs isolated from transfected COS-7 cells. Lane 1 shows no amplification product as expected in the PCR negative control; Lane 2 shows amplification of M6a complete CDS (circa 800 bp), 100ng of pDNA were used as template; Lane 3 shows negative amplification when 3µg total EVs protein isolated from COS-7 cells transfected with M6a-pDNA were used as template. To rule out PCR inhibitors, in lane 4 a pDNA spike in was added to the EV template (100ng pDNA+3µg total EVs protein). The expected product could be detected, proving no PCR inhibition. The gel image was not crop. The image was processed to show each band more clearly. Processing was applied equally across the entire image.

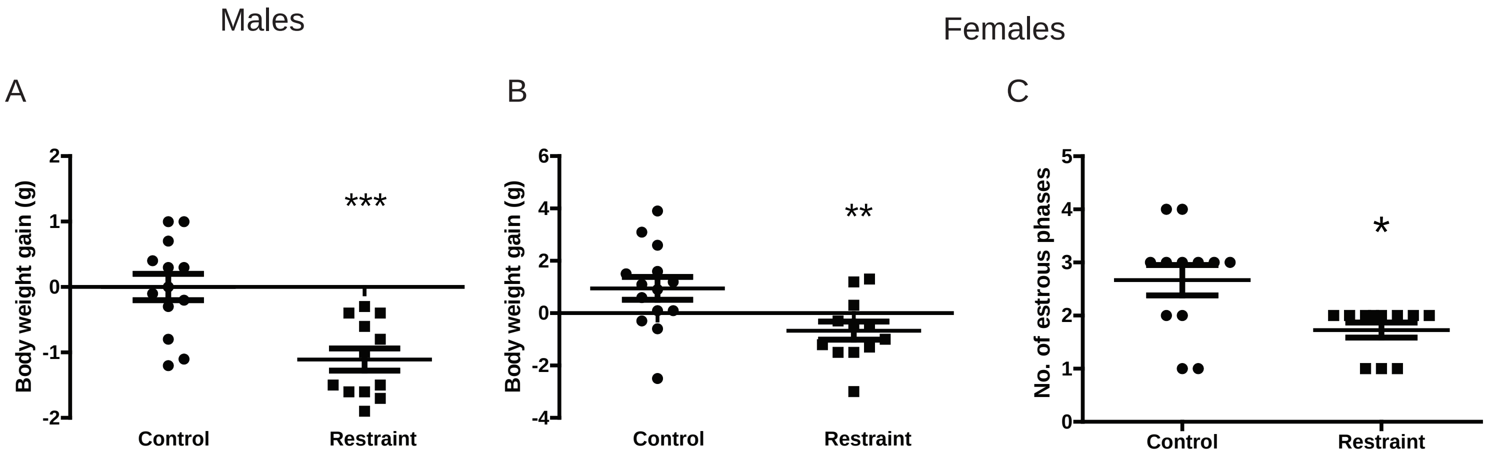

Fig S7. Effect of chronic restraint stress (CRS) on body weight gain and estrous phase entry. In both male (A) and female (B) there was a significant decrease in body weight gain after stress (***p<0.0005; t-Student test). In females subjected to CRS the number of estrous phases were also diminished (C) (*p<0.05; t-Student test). Each symbol represents one individual. Male n=13, Female n=12-14. Animals were weighted twice per week and female estrous phase was determined daily by vaginal smear analysis.

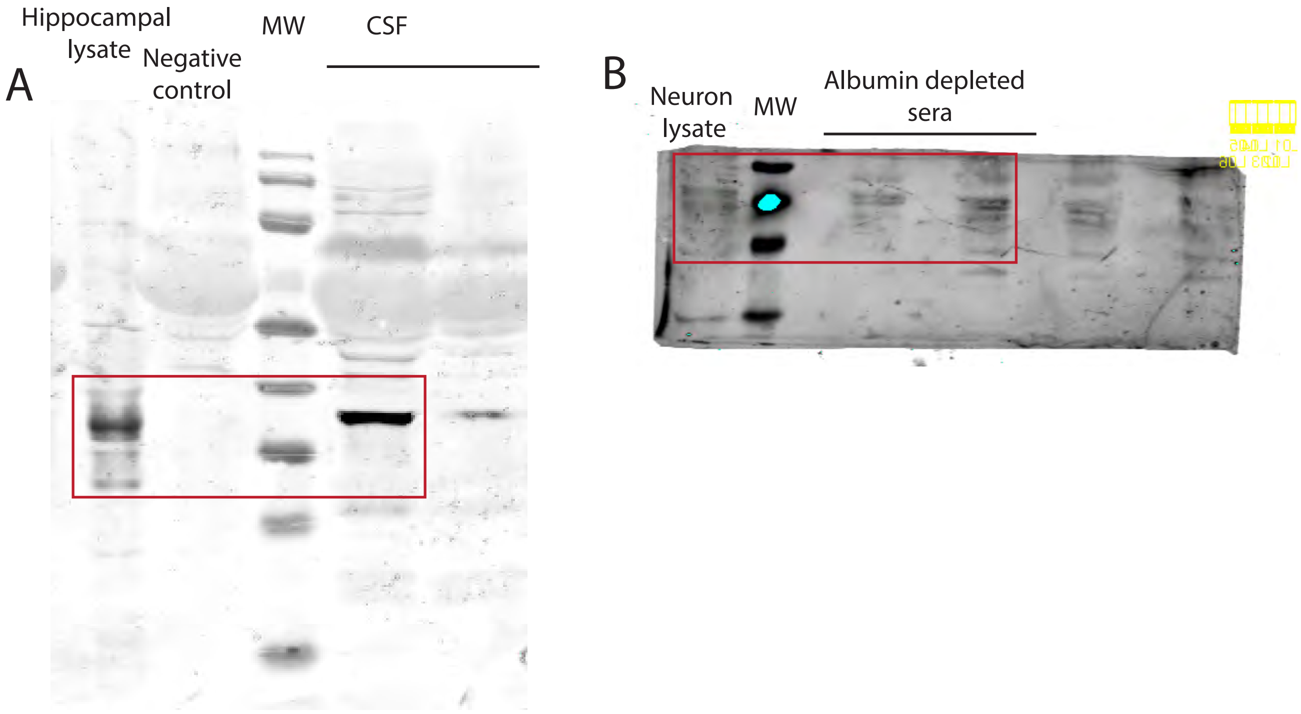

Figure S8. Original blots to Figure 1A, B. Blots for A: cerebrospinal fluid (CSF) and B: serum samples treated with anti-M6a. Red boxs indicate the areas included in the main paper.

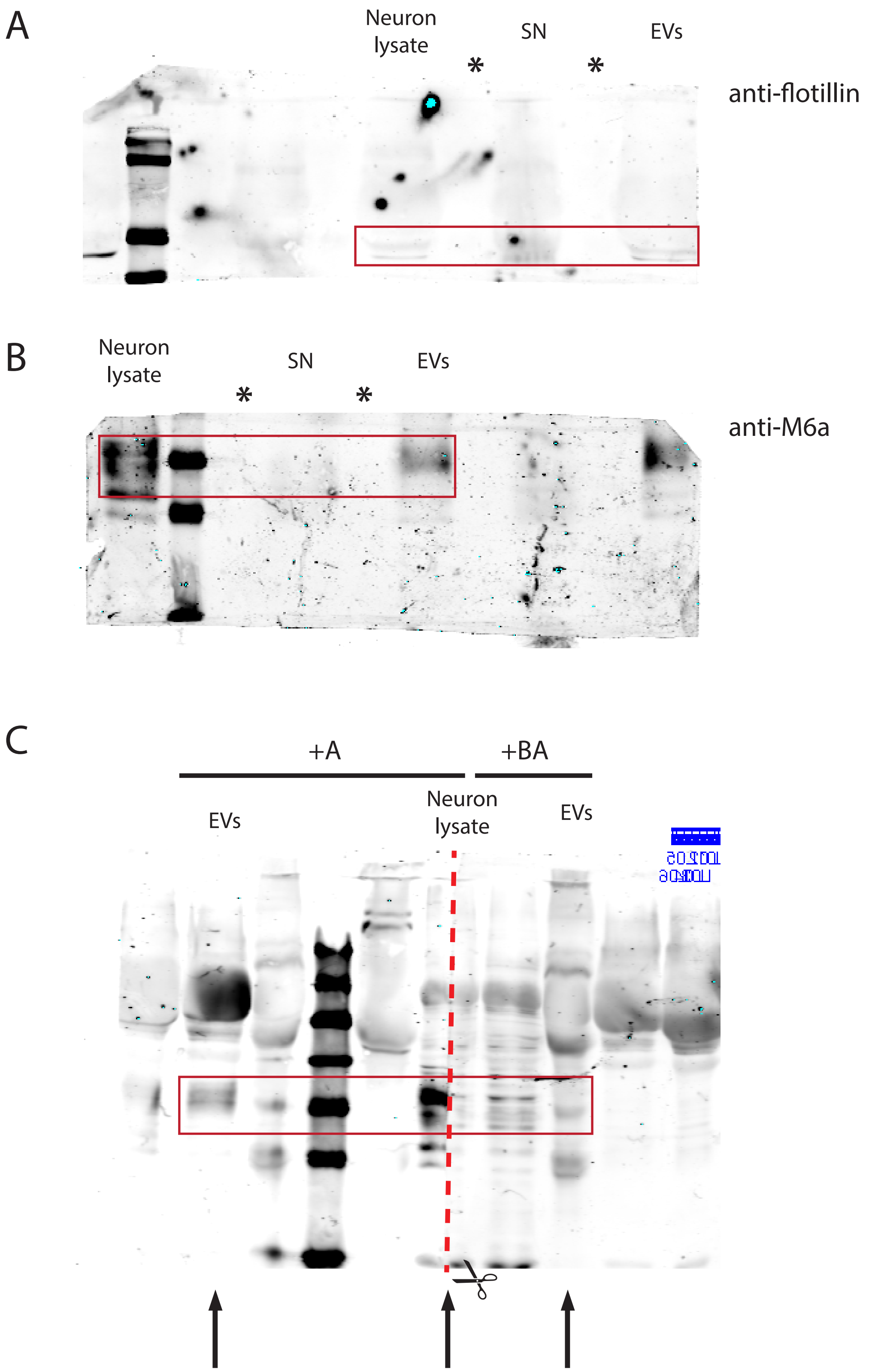

Figure S9. Original blots from Figure 2B, C. Membrane was treated with anti-flotillin (A) and anti-M6a (B) antibodies (A). Asterisks indicate empty lanes. (C) Blot with antibody treated (+BA) or not (+A) with the blocking peptide. Dotted line indicate where the membrane was cut for the different treatments. Arrows indicate lanes showed in the main paper. Red boxs indicate the areas included in the main paper.

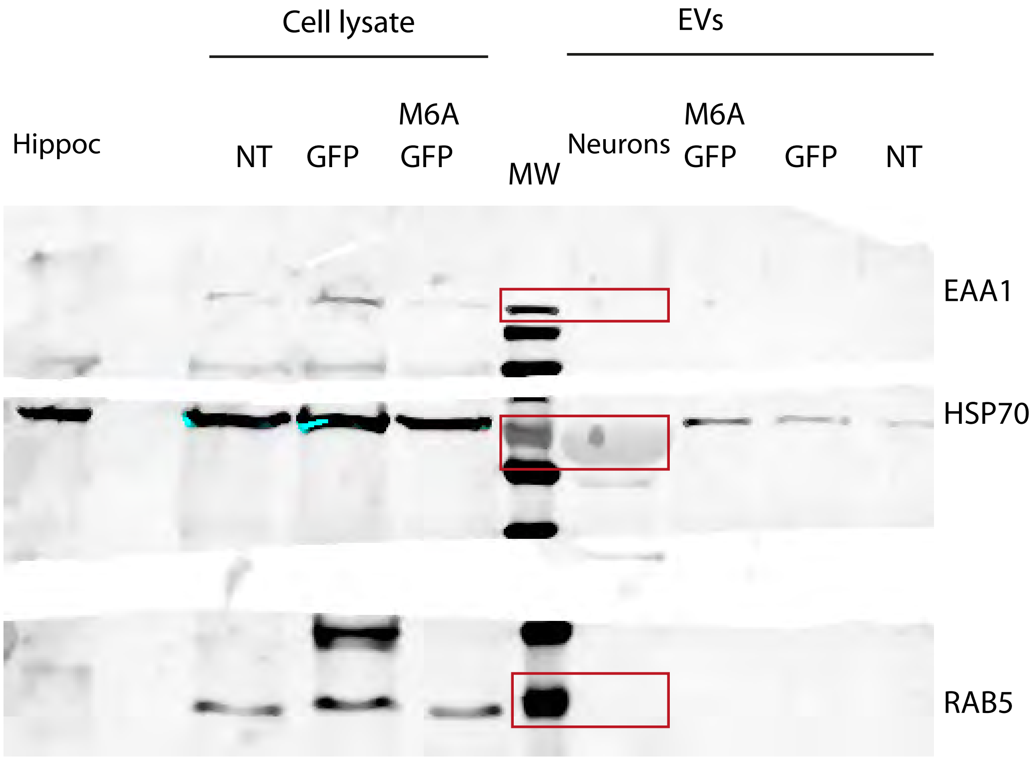

Figure S10. Original blots from Figure 2D. Membrane was treated with anti-EAAT1, anti-HSP70 and anti-RAB5. First lane: hippocampal tissue homogenate. NT: not transfected COS-7 cells, GFP: COS-7 cell transfected with GFP plasmid, M6a-GFP: COS-7 cells transfected with the plasmid coding the fusion protein M6a-GFP, MW: molecular weight marker. Red boxs indicate the areas included in the main paper.

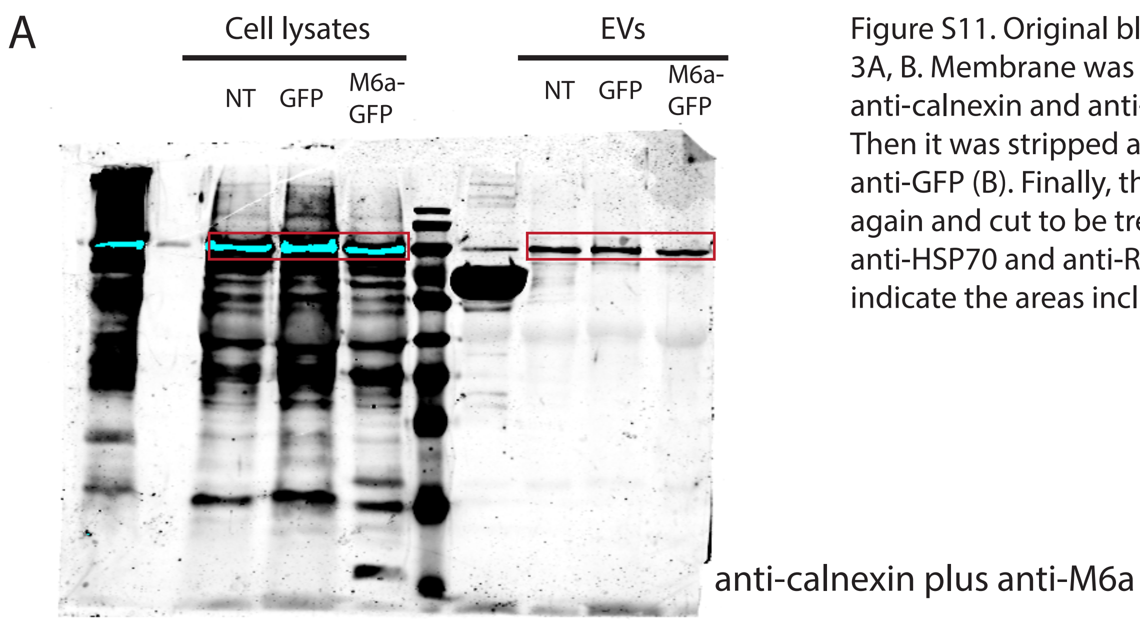

Figure S11. Original blots from Figure 3A, B. Membrane was treated with anti-calnexin and anti-M6a antibodies (A). Then it was stripped and treated with anti-GFP (B). Finally, the blot was stripped again and cut to be treated with anti-EAAT1, anti-HSP70 and anti-RAB5 (C). Red boxs indicate the areas included in the main paper.

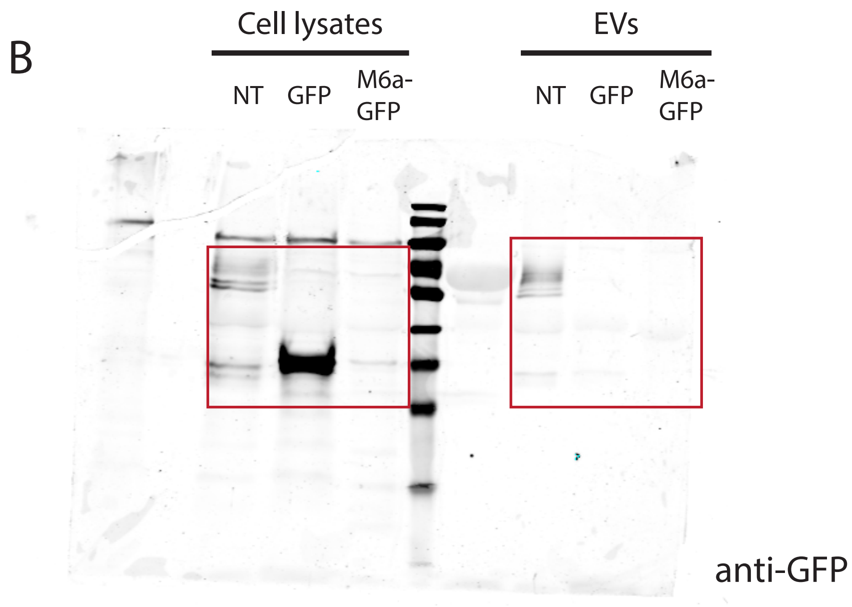

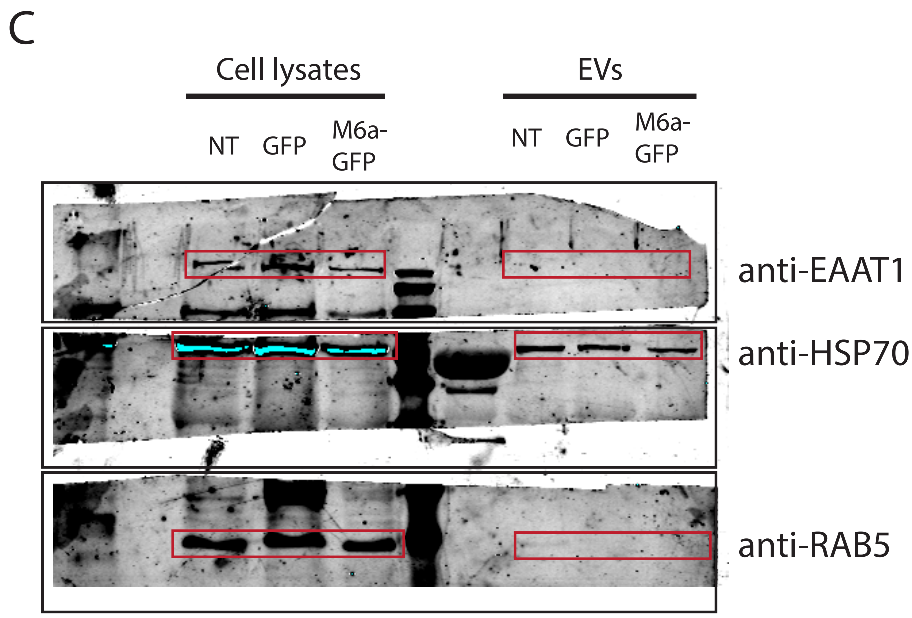

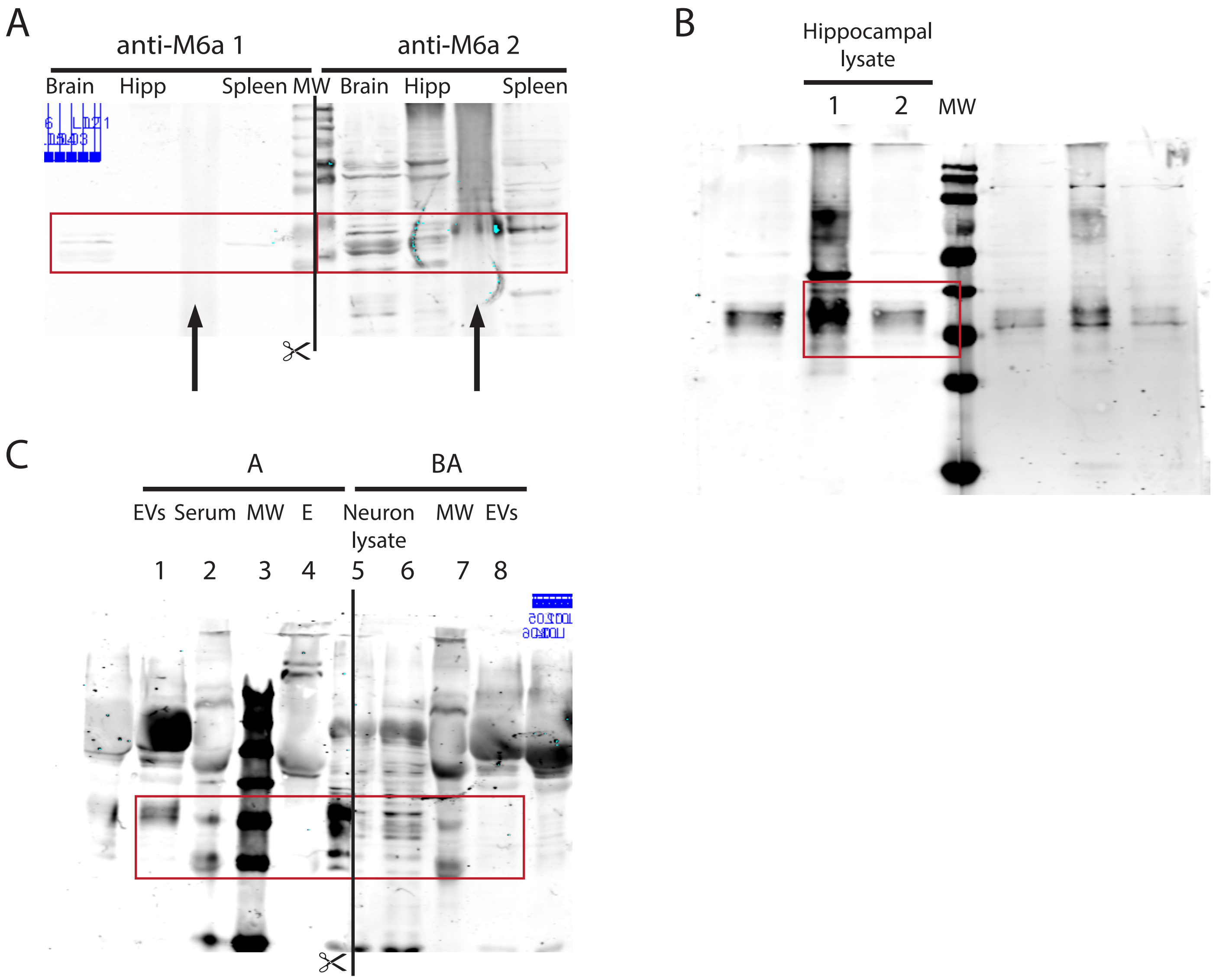

Figure S12. Original blots to Figures S1. **A.** Blot treated with 2 different anti-M6a antibodies. Arrows indicate lanes that were removed. Black line along the lane with molecular weight (MW) marker and scisors indicate where the membrane was cut. **B.** Blot with two hippocampal lysates. C. Blot with blocking peptide. Black line along the lane with neuronal lysate and scisors indicate where the membrane was cut. Red boxs indicate the areas showed in the main paper.
